# Pilot randomized trial of short-term changes in inflammation and lipid levels during and after aspirin and pravastatin therapy

**DOI:** 10.1186/s12978-019-0794-6

**Published:** 2019-09-02

**Authors:** Kerry S. Flannagan, Lindsey A. Sjaarda, Micah J. Hill, Matthew T. Connell, Jessica R. Zolton, Neil J. Perkins, Sunni L. Mumford, Torie C. Plowden, Victoria C. Andriessen, Jeannie G. Radoc, Enrique F. Schisterman

**Affiliations:** 1 Epidemiology Branch, Division of Intramural Population Health Research, Eunice Kennedy Shriver National Institute of Child Health and Human Development, National Institutes of Health, 6710B Rockledge Drive, MSC7004, Bethesda, MD 20894 USA; 2 Program in Reproductive and Adult Endocrinology, Eunice Kennedy Shriver National Institute of Child Health and Human Development, National Institutes of Health, Bethesda, MD USA; 3 Walter Reed National Military Medical Center, Bethesda, MD USA

**Keywords:** Pravastatin, Aspirin, Infertility treatment, Inflammation, Cholesterol, Overweight and obesity, Pilot study

## Abstract

**Background:**

Inflammation and elevated blood lipids are associated with infertility. Aspirin and statin therapy may improve infertility treatment outcomes among overweight and obese women with systemic inflammation, but little is known about the short-term effects of statins in this population. We conducted a pilot study of aspirin, pravastatin, or combined treatment among a group of overweight and obese, reproductive-aged women. Our goal was to characterize short-term changes in inflammatory and lipid biomarkers during and after treatment.

**Methods:**

In this open-label trial, women aged 18–40 years with a body mass index ≥25 kg/m^2^ were randomized to receive either 162 mg aspirin, 40 mg pravastatin, or both. The study medication was taken daily for 2 weeks, and participants were then followed for a two-week washout period. Participants provided blood samples at baseline, after the intervention period, and after the washout period. The outcomes were changes in biomarkers of inflammation and lipids measured in blood components at each timepoint.

**Results:**

Nine, 8, and 8 women were randomized to the aspirin, pravastatin, and combined arms, respectively. Analyses were conducted among 8, 7, and 7 women in the aspirin, pravastatin, and combined arms for whom biomarker data was available at baseline. High-sensitivity C-reactive protein (hsCRP) levels were lower after treatment in all arms and continued to decrease after washout in the pravastatin and combined arms. Results were consistent between the whole sample and women with baseline hsCRP between 2 and 10 mg/L. Low-density lipoprotein (LDL) cholesterol was lower after treatment in the pravastatin and combined arms and rose slightly after washout.

**Conclusions:**

Our results provide preliminary evidence that short-term aspirin and pravastatin therapy reduces hsCRP and LDL cholesterol among overweight and obese women of reproductive age, including those with low-grade inflammation. Because of these short-term effects, these drugs may improve infertility treatment outcomes in this population, which we will assess in a future randomized trial.

## Plain English summary

Low-grade inflammation is a condition in which the body produces low amounts of inflammatory factors for an extended period. Women with overweight/obesity often have both low-grade inflammation and elevated blood lipids, such as cholesterol and triglycerides. Inflammation and lipids may impair fertility because they are related to reproductive disorders. When these women undergo infertility treatment, taking aspirin and cholesterol-lowering statin drugs could improve their outcomes by reducing inflammation and cholesterol. However, because statins are not recommended during pregnancy, it is important to know whether their effects occur quickly after beginning treatment and persist after a woman stops taking the drug. This would indicate that short-term statin therapy stopped before pregnancy, along with aspirin, a common anti-inflammatory medication, could still support the events of early pregnancy. We conducted a preliminary study of short-term changes in inflammation and blood lipids during and after aspirin and pravastatin therapy in a group of overweight women.

We randomly assigned women to take either 162 mg aspirin, 40 mg pravastatin, or both daily for 2 weeks. We measured inflammation and lipids in blood samples before and after the 2 weeks when participants took the medication, and 2 weeks after they stopped.

All women had lower inflammation after treatment, and women taking pravastatin had lowered inflammation 2 weeks post-treatment. These women also had lower cholesterol levels after treatment. Our results indicate that aspirin and pravastatin might improve infertility treatment outcomes among overweight women with low-grade inflammation, which we intend to test in a larger trial.

## Background

Inflammation is associated with multiple types of reproductive disorders and infertility [1–3]. Higher lipid levels are also associated with lower oocyte quality [4, 5] and lower likelihood of pregnancy [6, 7].

Interventions aimed at reducing systemic inflammation and blood lipid levels might be effective at improving outcomes of first-line infertility treatments such as ovulation induction (OI) and/or intrauterine insemination (IUI), which currently have relatively low success rates [8–11]. In the Effects of Aspirin in Gestation and Reproduction (EAGeR) Study, inflammation measured by high-sensitivity C-reactive protein (hsCRP) was associated with lower fecundity [12]. However, low-dose aspirin therapy restored normal pregnancy and live birth rates only among lean women with low-grade inflammation [12]. For women with higher adiposity, a higher dose of aspirin may be necessary, and a dual therapy targeting both inflammation and lipid levels may provide additional benefit and be needed to improve reproductive outcomes.

In addition to aspirin, statin drugs may be beneficial in addressing infertility. Statins have lipid-lowering effects, and are also anti-inflammatory agents [13–15]. However, while the long-term effects of these drugs are well studied [13, 16], there is very little information on their effects in the short term. Because statins are contraindicated in pregnancy due to potential teratogenic effects [17], understanding the timing of anti-inflammatory and lipid-lowering actions during short-term statin therapy is crucial for determining how these drugs might be used in infertility treatment. To address this gap, we conducted a pilot randomized trial of aspirin and pravastatin therapy among overweight and obese, reproductive-aged women. Our aim was to obtain preliminary information on short-term changes in inflammatory and metabolic biomarkers during and after cessation of treatment.

## Methods

The study design was an unblinded, randomized trial. Participants were women seeking infertility evaluation at the Walter Reed National Military Medical Center (WRNMMC) in Bethesda, MD. Study personnel approached women at their initial clinic visit regarding the study and assessed eligibility criteria. Eligible women were between 18 and 40 years old; had a body mass index (BMI) ≥25 kg/m^2^; had a negative urine hCG pregnancy test at screening; were not currently using aspirin, statins, other cholesterol medications, non-steroidal anti-inflammatory drugs, or oral contraceptives; free of major chronic health conditions; and did not have any other conditions for which aspirin or statins would be contraindicated. Women who met these criteria were invited to enroll. To ensure enrollment of enough women with hsCRP between 2 and 10 mg/L, indicating low-grade inflammation, the target sample size was 39; however, enrollment was stopped at 27 due to practical considerations related to the larger trial. All participants provided informed written consent for screening and study procedures. The study was approved by the institutional review board at WRNMMC, where all study procedures and biomarker measurements took place.

Enrolled women were randomized with a computer algorithm to receive either 162 mg aspirin, 40 mg pravastatin, or both. The aspirin dosage is based on previous data showing lowered inflammatory markers at this dose [18], and the dosage of pravastatin was chosen because it is used in normal therapy. A block-randomized list of treatment assignments was generated and written in sealed envelopes prior to study initiation, and the next envelope in order was chosen as each participant was enrolled. The study medication was taken daily by mouth for 2 weeks at no specific time of day. Following this period, women were followed for an additional 2-week washout period. The treatment period was designed to mimic a realistic course of treatment in our future trial, where study medications would be taken from the beginning of the menstrual cycle until ovulation 2 weeks later. The washout period was designed to determine whether effects of the medications would last throughout events of pregnancy establishment during the 2 weeks following ovulation and treatment cessation. In addition to the study intervention, participants were asked to abstain from intercourse while taking the study medication. Participants provided blood samples before and after the intervention period, and after the washout period (baseline, week 2, and week 4, respectively). Reproductive history characteristics were obtained at baseline.

The outcomes of interest were inflammatory and lipid biomarkers measured in blood components at baseline, week 2, and week 4. The inflammatory biomarkers included serum hsCRP, serum interleukin-1β (IL-1), and serum interleukin-6 (IL-6). HsCRP was measured by immunoturbidimetric assay (Roche Diagnostics, Indianapolis, IN), IL-1 was measured by enzyme-linked immunosorbent assay (Quest Diagnostics, Baltimore, MD, USA), and IL-6 was measured by chemiluminescent immunoassay (Siemens Healthcare Global, Malvern, PA). Lipids include serum total cholesterol, serum low-density lipoprotein (LDL) cholesterol, serum high-density (HDL) lipoprotein cholesterol, and serum triglycerides. All lipids were measured by homogenous enzymatic colorimetric assay (Roche Diagnostics, Indianapolis, IN). We compared medians and ranges of the biomarkers at the three timepoints with respect to treatment arm. We also examined individual trajectories of hsCRP among women with baseline hsCRP between 2 and 10 mg/L, indicating chronic, low-grade inflammation.

## Results

Twenty-seven women were recruited and 2 withdrew before randomization. The final randomized sample included 9, 8, and 8 women in the aspirin, pravastatin, and combined arms, respectively. After withdrawals, the final numbers of women with available biomarker measurements at baseline, week 2, and week 4 were 22, 19, and 11, respectively. The mean (± SD) age of randomized participants was 31.8 ± 5.1 years and mean (± SD) BMI was 31.4 ± 5.9 kg/m^2^. The treatment arms were balanced with respect to most baseline demographic and reproductive characteristics, although the median age of women in the aspirin arm was somewhat lower compared to the other two groups (Table 1).

**Table 1:** Baseline characteristics of participants by assigned treatment group

	Treatment group		
	Aspirin only (*N* = 9)	Pravastatin only (*N* = 8)	Aspirin and pravastatin (*N* = 8)
Characteristic	Median (range) or N	Median (range) or N	Median (range) or N
Age (y)	29 (23, 41)	33 (29, 39)	34 (24, 40)
Body mass index (kg/m^2^)	28.3 (26.1, 35.5)	29.3 (25.8, 46.7)	31.0 (26.6, 48.1)
Polycystic ovary syndrome	3	1	2
Prior pregnancies	1 (0, 3)	1.5 (0, 3)	0.5 (0, 3)
Prior fullterm births	0 (0, 2)	0 (0, 1)	0 (0, 2)
Prior pregnancy losses or terminations	1 (0, 1)	0.5 (0, 3)	0 (0, 2)

**Fig. 1:**
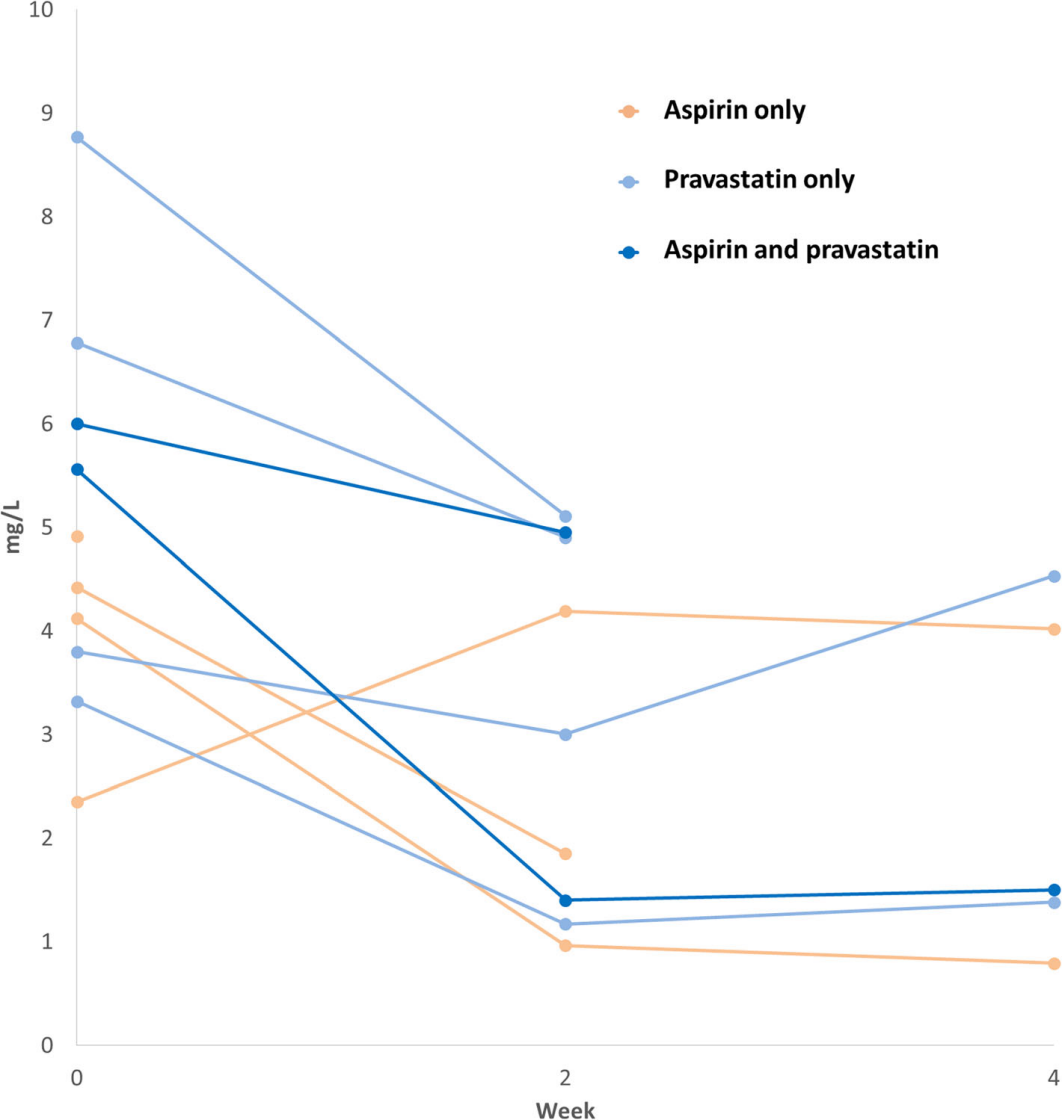
Trajectories of hsCRP among women with baseline hsCRP between 2 and 10 mg/L. Each series represents one participant; data are individual measurements of hsCRP in mg/L. Women were randomized to receive either daily aspirin, pravastatin, or both from baseline to week 2, followed by a washout period from week 2 to week 4

Compared with baseline, median hsCRP was lower after 2 weeks of treatment in all three study arms (Table 2); these differences were most pronounced in the aspirin and combined treatment groups. At week 4, median hsCRP in the aspirin group rose and was again similar to baseline, whereas among the pravastatin and combined groups it continued to decrease. Among women with baseline hsCRP between 2 and 10 mg/L (*N* = 10), hsCRP levels decreased from baseline to week 2 in 2 of 3 women in the aspirin group, 4 of 4 women in the pravastatin group, and 2 of 2 women in the combined group (Fig. 1). Five of these women had biomarker measurements available at week 4 (2 in the aspirin group, 2 in the pravastatin group, and 1 in the combined group). One woman in the pravastatin group had higher hsCRP at week 4 compared to week 2; the other women had no change.

**Table 2:** Medians of inflammatory and cardiometabolic biomarkers over time by assigned treatment

	Treatment group					
	Aspirin only		Pravastatin only		Aspirin and pravastatin	
Biomarker	N	Median (range)	N	Median (range)	N	Median (range)
hsCRP (mg/L)						
Baseline	8	2.1 (0.2, 4.9)	7	3.3 (0.4, 8.8)	6	3.6 (0.3, 12.0)
Week 2	7	1.0 (0.2, 4.2)	7	2.9 (0.3, 5.1)	5	1.4 (0.3, 16.2)
Week 4	3	2.3 (0.8, 4.0)	4	1.1 (0.6, 4.5)	4	0.9 (0.3, 11.1)
IL-1 (pg/mL)						
Baseline	8	3.9 (3.9, 3.9)	7	3.9 (3.9, 4.0)	7	3.9 (3.9, 4.0)
Week 2	7	3.9 (3.9, 3.9)	7	3.9 (3.9, 4.0)	5	3.9 (3.9, 4.0)
Week 4	3	3.9 (3.9, 4.0)	4	4.0 (3.9, 4.0)	4	3.9 (3.9, 4.0)
IL-6 (pg/mL)						
Baseline	8	1.9 (0.7, 3.0)	7	3.4 (0.7, 41.1)	6	3.2 (0.7, 15.4)
Week 2	7	2.0 (0.7, 3.1)	7	3.4 (0.7, 8.7)	5	2.4 (0.7, 7.6)
Week 4	3	2.8 (0.7, 3.1)	4	2.1 (0.7, 5.5)	4	2.4 (0.7, 10.2)
Total cholesterol (mg/dL)						
Baseline	8	192 (120, 238)	6	180 (159, 256)	7	166 (145, 188)
Week 2	6	183 (153, 209)	5	173 (124, 224)	5	148 (137, 191)
Week 4	3	186 (157, 232)	4	179 (140, 190)	3	147 (116, 154)
LDL cholesterol (mg/dL)						
Baseline	8	125 (79, 189)	6	115 (92, 145)	7	102 (7, 122)
Week 2	6	114 (83, 143)	5	87 (58, 130)	5	78 (60, 101)
Week 4	3	113 (104, 189)	4	104 (73, 130)	3	90 (52, 101)
HDL cholesterol (mg/dL)						
Baseline	8	54 (30, 83)	6	61 (46, 87)	7	61 (33, 82)
Week 2	6	64 (34, 79)	5	64 (51, 82)	5	60 (36, 75)
Week 4	3	40 (38, 62)	4	60 (47, 72)	3	59 (32, 60)
Triglycerides (mg/dL)						
Baseline	8	111 (33, 311)	6	87 (31, 146)	7	75 (73, 121)
Week 2	6	71 (37, 229)	5	105 (48, 138)	5	98 (54, 166)
Week 4	3	92 (31, 97)	4	73 (35, 142)	3	113 (73, 206)

There were no differences in IL-1 concentrations over time in any group (Table 2). In the aspirin group, IL-6 concentrations were similar at baseline and week 2 but rose by week 4. IL-6 in the pravastatin group also did not change appreciably between baseline and week 2 but decreased between weeks 2 and 4. In the combined group, median IL-6 decreased from baseline to week 2 and remained similar at week 4.

Median total cholesterol concentrations were similar throughout the study in the aspirin and pravastatin groups. In the combined group, total cholesterol was lower at weeks 2 and 4 compared to baseline. LDL cholesterol was consistent at all time points in the aspirin group. In the pravastatin and combined groups, median LDL cholesterol decreased between baseline and week 2, then rose slightly between weeks 2 and 4. HDL cholesterol lowest at week 4 in the aspirin group but did not change in the pravastatin and combined groups. Serum triglyceride trajectories were inconsistent between all treatment arms. In the aspirin group, the median concentration initially fell and then rose across the 3 timepoints, whereas the pravastatin group experienced the opposite pattern. In the combined group, median triglyceride concentrations rose consistently across follow-up.

## Discussion

In this pilot randomized trial of short-term treatment with aspirin, pravastatin, or both, we found that hsCRP was reduced after 2 weeks of medication in all treatment arms. Two weeks after treatment cessation, hsCRP remained below baseline levels among participants in the groups receiving pravastatin. Results among women with elevated baseline hsCRP were consistent with results among the whole sample.

Our results are consistent with previous research indicating that statins have anti-inflammatory effects [13, 19], including studies conducted among women of reproductive age with polycystic ovary syndrome [20, 21]. Most work in this area has focused on longer-term statin use; however, one previous study assessed short-term treatment and inflammation [15]. In this trial, 40 men and women with elevated LDL cholesterol received either simvastatin for 2 weeks followed by placebo for 2 weeks or vice versa. During statin treatment, mean hsCRP decreased from 2.55 mg/L at baseline to 1.60 mg/L after 2 weeks, while there was no decrease during the placebo periods. These results are consistent with our findings that statin-mediated changes in inflammation occur in a relatively short timeframe. We also found evidence for continued suppression of inflammation 2 weeks after statin cessation, in contrast with a previous study of hyperlipidemic participants in which CRP rose within a week of stopping pravastatin [14].

Our results provide preliminary evidence for short-term anti-inflammatory effects of statins in a population of reproductive-aged overweight women with low-grade inflammation, a group who might benefit from the use of statins in conjunction with infertility treatments such as OI/IUI. None of the participants in this study reported adverse effects of the study medications such as bleeding or muscle pain, suggesting that these treatments can be well-tolerated. Since statins are contraindicated in pregnancy because of possible teratogenicity [17], the timing of inflammation dynamics during and after statin therapy have potential implications for its use in this context. The utility of statin drugs may be related to whether short-term therapy, stopped at or before the first indication of pregnancy, can reduce inflammation during preconception and early pregnancy.

We also found that LDL cholesterol was lower after treatment among both groups receiving pravastatin, but rose after treatment cessation. The finding that statins lower LDL cholesterol within 2 weeks is consistent with previous work [15]. However, in a study of six-week pravastatin therapy among hyperlipidemic participants, there was no change in LDL 2 week after stopping treatment [14]. Potential explanations for this discrepancy include the duration of the statin treatment, demographic and health-related differences between the study populations, or chance.

One of the strengths of this study is its randomized design. The study also provides novel information on the timing of statins’ anti-inflammatory effects among overweight, reproductive-aged women with low-grade inflammation. Understanding these effects in this population is important, since the use of statins to address inflammation and elevated lipids could improve infertility treatment outcomes in these women. One of the study’s primary weaknesses was the high rate of loss to follow-up, which could have biased our results if dropout was related to inflammation or lipid levels. Because we were not able to screen for hsCRP as an inclusion criterion, the number of women with elevated hsCRP at baseline was small. Finally, we did not collect fasting blood samples, which could have induced extraneous variation in measurements of some blood lipids.

## Conclusions

Overall, our findings provide preliminary evidence that short-term treatment with pravastatin and aspirin may reduce inflammation and LDL cholesterol in women seeking infertility treatment. We are currently in the process of designing a larger trial of medication use to improve fertility treatment outcomes in a similar population of women with chronic, low-grade inflammation, including those with and without obesity. In the long-term, if statins and aspirin prove beneficial for women with chronic inflammation who wish to become pregnant, these drugs could be added to clinical practice as part of first-line fertility treatments such as OI/IUI, in order to improve the success rates of these procedures.

## Data Availability

The datasets used and/or analyzed during the current study are available from the corresponding author on reasonable request.

## Abbreviations

BMI: Body mass index
EAGeR: Effects of Aspirin in Gestation and Reproduction Study
HDL: High-density lipoprotein
hsCRP: High-sensitivity C-reactive protein
IL-1: Interleukin-1
IL-6: Interleukin-6
IUI: Intrauterine insemination
LDL: Low-density lipoprotein
OI: Ovulation induction
SD: Standard deviation
WRNMMC: Walter Reed National Military Medical Center
